# Calibration-Less Multi-Coil Compressed Sensing Magnetic Resonance Image Reconstruction Based on OSCAR Regularization

**DOI:** 10.3390/jimaging7030058

**Published:** 2021-03-19

**Authors:** Loubna El Gueddari, Chaithya Giliyar Radhakrishna, Emilie Chouzenoux, Philippe Ciuciu

**Affiliations:** 1 NeuroSpin, CEA, Université Paris-Saclay, 91191 Gif-sur-Yvette, France; loubna.elgueddari@gmail.com (L.E.G.); chaithyagr@gmail.com (C.G.R.); philippe.ciuciu@cea.fr (P.C.); 2 Parietal, Inria, 91120 Palaiseau, France; 3 OPIS, Inria, Université Paris-Saclay, 91190 Gif-sur-Yvette, France

**Keywords:** compressed sensing, parallel MRI, non-Cartesian acquisition, cluster norm, proximal algorithms

## Abstract

Over the last decade, the combination of compressed sensing (CS) with acquisition over multiple receiver coils in magnetic resonance imaging (MRI) has allowed the emergence of faster scans while maintaining a good signal-to-noise ratio (SNR). Self-calibrating techniques, such as ESPiRIT, have become the standard approach to estimating the coil sensitivity maps prior to the reconstruction stage. In this work, we proceed differently and introduce a new calibration-less multi-coil CS reconstruction method. Calibration-less techniques no longer require the prior extraction of sensitivity maps to perform multi-coil image reconstruction but usually alternate estimation sensitivity map estimation and image reconstruction. Here, to get rid of the nonconvexity of the latter approach we reconstruct as many MR images as the number of coils. To compensate for the ill-posedness of this inverse problem, we leverage structured sparsity of the multi-coil images in a wavelet transform domain while adapting to variations in SNR across coils owing to the OSCAR (octagonal shrinkage and clustering algorithm for regression) regularization. Coil-specific complex-valued MR images are thus obtained by minimizing a convex but nonsmooth objective function using the proximal primal-dual Condat-Vù algorithm. Comparison and validation on retrospective Cartesian and non-Cartesian studies based on the Brain fastMRI data set demonstrate that the proposed reconstruction method outperforms the state-of-the-art ($\ell_{1}$-ESPIRiT, calibration-less AC-LORAKS and CaLM methods) significantly on magnitude images for the T1 and FLAIR contrasts. Additionally, further validation operated on 8 to 20-fold prospectively accelerated high-resolution ex vivo human brain MRI data collected at 7 Tesla confirms the retrospective results. Overall, OSCAR-based regularization preserves phase information more accurately (both visually and quantitatively) compared to other approaches, an asset that can only be assessed on real prospective experiments.

## 1. Introduction

Compressed sensing (CS) [1,2,3,4] has made a breakthrough in the MR community and now in clinics with recent Food and Drug Administration (FDA) approval [5] as it provides ways to drastically shorten scan times especially when adopting non-Cartesian sampling schemes (radial, Propeller, spiral, Sparkling) [6,7,8,9,10,11] in the k-space. Non-Cartesian sampling patterns actually offer many advantages such as robustness to motion or better sampling efficiency [6,7,8,10,11]. For these reasons, non-Cartesian 2D acquisitions make the use of higher acceleration factors feasible as compared to Cartesian sampling. In the race for rapid high-resolution scan, preserving a high signal-to-noise ratio (SNR) is mandatory. Two ingredients may contribute to achieving this goal—on the one hand, moving to 3D non-Cartesian imaging provides an increased SNR [11,12]. On the other hand, CS k-space acquisition is usually combined with multi-receiver coils [13] allowing for the acquisition of multiple and complementary information over a network of sensors. In this paper, we focus on this second point.

CS reconstruction of MR images from multi-coil under-sampled k-space data has generated tens of contributions over the last decade. Although the landscape of reconstruction methods is predominantly dominated by Cartesian methods such as SAKE [14] or P-LORAKS [15], which relies on low-rank constraints in the k-space, the room for non-Cartesian reconstruction techniques is left pretty empty. In the latter setting, MR image reconstruction actually consists in solving an inverse problem and amounts to minimizing an objective function where the data consistency term is balanced with a sparse prior (e.g., the $\ell_{1}$-norm) over a given domain (e.g., the image gradient or a wavelet transform) [16,17]. This prior enforces the image sparsity in this transformed domain. In that context, two types of reconstruction methods exist for non-Cartesian acquisitions: self-calibrating and calibration-less approaches.

Self-calibrating methods are based on the reconstruction of the sensitivity-combined image, which requires the explicit knowledge of sensitivity maps. Therefore such methods propose the extraction of sensitivity profiles associated with the multiple coils prior to the reconstruction itself. As the sensitivity profiles are spatially smooth they can be extracted from the center of k-space, which is usually densely sampled (along a variable density as required by CS for optimal acceleration [18,19]). The extraction of sensitivity maps has been the matter of several works (cf. [20,21,22]). Once these profiles are estimated, they can be plugged in a sensitivity encoding (SENSE)-based formulation of the multi-coil CS reconstruction problem [16,17]. Alternatively, one can jointly solve image reconstruction and coil sensitivity estimation as a blind bi-linear inverse problem, as done for instance in JSENSE [23], its variant [24] and iSENSE [25]. The image and sensitivity maps are typically regularized using different regularization terms such as sparsity in a wavelet basis for the image and low rank constraint (using the nuclear norm) for the sensitivity maps. Overall in these methods, the computation cost becomes prohibitively expensive for high-resolution imaging as one has to perform alternate minimization of the global objective function with respect to the image and sensitivity maps. Moreover, the non-convexity of the global cost function makes the final solution dependent on its initialization even though each subproblem is convex. The difficulty lies in the fact that certain local minimizers may correspond to poorer image quality and once being trapped in them, it is impossible to visit other basins of attraction unless restarting the algorithm. A recent extension of these works that still proceeds to alternate minimization with respect to image and sensitivity maps has been proposed in [26] with some additional physical constraint on the sensitivity maps. We note, however, that the authors of this contribution qualified their work as a calibration-less CS reconstruction method for multi-coil MRI data. We will adopt a different meaning for this terminology in the next paragraph.

### 1.1. Related Works

Instead of considering a 2-step procedure, calibration-less MR image reconstruction methods such as CalM (Calibration-less Multi coil) [27] came up with the idea of reconstructing one image per channel and enforcing some common prior knowledge between all images through for instance structured sparsity. Two different regularization terms have been tested so far, group-sparsity in the wavelet domain [27,28] and patch-based *local* low-rank in the image domain (CLEAR) [29]. In terms of group-sparsity, the group-LASSO is likely the most convenient and efficient mixed-norm used for MR image reconstruction in the multi-coil CS framework [27,28]. Although Chun et al. [28] demonstrate that promoting structured sparsity over the channels actually improves exact recovery guarantees in the multi-coil CS framework (One can lower the number of k-space samples to get perfect image reconstruction in the noise-free case as multiple k-space are collected over the receiver channels.), the group-LASSO regularization is over-simplistic. It actually assumes that the same image support holds in an appropriate transform domain (e.g., total variation, wavelets, frames) across all receiver channels. As such, it neglects the SNR fluctuations that exist between the multiple receivers of a given coil in any given region of the organ to be probed (e.g., the brain).

### 1.2. Our Contributions

In this work, we propose a new calibration-less reconstruction method that goes beyond the group-LASSO penalty and takes advantage of the redundant information provided by each receiver coil. As a reminder, group-LASSO regularization considers the information to be spread uniformly and thus uses the same weight for all channels in the present context. In contrast, our approach promotes structured sparsity across coils using a clustering and shrinkage algorithm, namely the octagonal shrinkage and clustering algorithm for regression (OSCAR) norm [30,31]. OSCAR regularization actually relies on a combination of a $\ell_{1}$ and pairwise $\ell_{\infty }$ norms that enables spatial clustering across receiver coils and thus implements an adaptive structured sparsity regularization. Practically, OSCAR may automatically assign *coil* and *location*-specific weights depending on their local SNR in order to get the highest image quality at the reconstruction stage. Consequently, with OSCAR regularization no receiver channel is discarded, they are just all ranked in terms of information content and treated accordingly. Interestingly, the OSCAR norm has a closed form proximity operator and as such is amenable in any nonsmooth proximity based optimization algorithm. In this work, we derive four versions of OSCAR regularization models and identify which one achieves the best trade-off between computational complexity and image quality for MR image reconstruction.

### 1.3. Outline of the Paper

The rest of the paper is organized as follows—in Section 2, we define the general formulation of the calibration-less MR image reconstruction problem. Then we detail the proximal primal-dual optimization algorithm we used to solve this problem. In Section 3, we recall the formulation of the OSCAR structured penalty. We then explore four variations of OSCAR-based regularization for the image reconstruction problem at hand and outline their differences for parallel computation purposes. In Section 4, we introduce the experimental setup that we used for retrospective (artificial data under-sampling using handcrafted Cartesian and non-Cartesian sampling masks) and prospective validation (real accelerated acquisitions at 7 Tesla) of the proposed approach. In particular, we rely on the publicly available brain fastMRI data set for the retrospective study and we collected our own ex vivo human brain data for the prospective one. In Section 5 we present the results of our benchmark of calibration-less CS reconstruction methods (AC-LORAKS, CaLM, OSCAR) and some comparisons with self-calibrating method ($\ell_{1}$-ESPIRiT). In Section 6, we discuss the pros and cons of the proposed approach in comparison with its competitors. Conclusions and perspectives are drawn in Section 7.

## 2. Problem Statement

### 2.1. Notation and Definitions

In the following, we will denote vectors with bold letters, for example, $v=\left[v_{1},\cdots ,v_{p}\right]\in C^{p}$ a *p*-size complex-valued vector. Matrices are denoted using bold upper case letters (e.g., ***A***). The transpose of a matrix ***A*** is denoted by $A^{⊤}$, its Hermitian transpose by $A^{*}$, its spectral norm by ‖|A|‖, and its Frobenius norm by ${\left\|A\right\|}_{2}$.

Let $\Gamma_{0}\left(C^{p}\right)$ the set of convex, proper, lower semi-continuous functions [32] on $C^{p}$ taking values on R∪+∞. The proximity operator of a function $g\in \Gamma_{0}\left(C^{p}\right)$ at $\in C^{p}$ is uniquely defined as [33]:

(1) $${\mathrm{prox}}_{g}\left(z\right)=\underset{v\in C^{p}}{\arg\min}\frac{1}{2}{\left\|z-v\right\|}^{2}+g\left(v\right).$$

### 2.2. General Problem Formulation

Let us focus on the problem of MR image reconstruction in the multi-receiver coil acquisition. We set *n* the image resolution in pixels and $N=n^{2}$ the image size, *L* the number of coils used to acquire the NMR signal and *M* the number of k-space measurements per coil, with M<N. For the sake of compactness, we denote the complete data set $Y=[y_{1},\ldots y_{L}]\in C^{M\times L}$ which stacks $y_{\ell }\in C^{M}$ the k-space samples collected in the *ℓ*th coil. Our goal is to recover *L* coil-specific complex-valued MR images that will be eventually combined to form a single full field-of-view (FOV) image. The way this combination is achieved will be clarified hereafter (especially for phase information).

In what follows, we do not take off-resonance effects and field inhomogeneities into account (see for instance [34] to handle fast conjugate phase reconstruction in the single channel case.) and assume that the simple 2D discrete Fourier operator states for the forward model relating the k-space measurements to the unknown MR images. Actually, this operator heavily depends on the retained sampling strategy during the acquisition process. In the Cartesian sampling case, $F_{M}=MF$, with ***F*** the 2D fast Fourier transform (FFT) and ***M*** the binary under-sampling mask defined over the discrete grid where each non-zero entry in ***M*** selects a row in ***F***. In non-Cartesian settings, ***F*** refers to non-equispaced or nonuniform FFT [35,36] and ***M*** stands for the discrete support of the k-space location measurements. Each coil measurement $y_{\ell }$, with ℓ∈{1,…,L}, is furthermore affected by an additive circular complex i.i.d. zero-mean Gaussian noise of variance $\sigma_{\ell }^{2}$, which can be characterized by a separate scan (without RF pulse) considering the same bandwidth ${\mathrm{BW}}_{\mathrm{read}}$ as the prospectively accelerated acquisition. For the sake of simplicity, we do not model here any potential between-coil covariance structure **∑**, which is thus assumed diagonal, $\sum =I_{L}$.

The goal is then to retrieve, from the noisy under-sampled data ${\left(y_{\ell }\right)}_{1\leq \ell \leq L}$, *L* MR images stacked in $X=[x_{1},\ldots ,x_{L}]\in C^{N\times L}$ such that each $x_{\ell }\in C^{N}$ is associated with the $\ell^{\mathrm{th}}$ coil of the phased array. We propose to solve this ill-posed inverse problem by adopting a variational penalized formulation, which consists in minimizing the following criterion:

(2) $$\hat{X}=\underset{X\in C^{N\times L}}{\arg\min}\{\sum_{\ell =1}^{L}\frac{1}{2\sigma_{\ell }^{2}}{∥F_{M}x_{\ell }-y_{\ell }∥}_{2}^{2}+g\left(\Psi X\right)\;\}.$$

This formulation enables the use of over-complete dictionaries [37,38]. Above, $g\in \Gamma_{0}\left(C^{N_{\Psi }\times L}\right)$ is a regularization function composed with a linear operator $\Psi \in C^{N_{\Psi }\times N}$, $N_{\Psi }\geq 1$, with the aim to enforce sparsity of the solution within a given multiscale decomposition (e.g., wavelet transform). We will assume that **Ψ** decomposes the stack of *L* images $X\in C^{N\times L}$ into a stack of coefficients $\Psi X\in C^{N_{\Psi }\times L}$ with *C* scales. Each scale c∈{1,⋯,C} is composed of $S_{c}$ sub-bands. Each sub-band $s\in \{1,\cdots ,S_{c}\}$ has $K_{s(c)}$ coefficients, so that finally $N_{\Psi }=\sum_{c=1}^{C}\sum_{s=1}^{S_{c}}K_{s(c)}$. For the sake of simplicity, in what follows we assume that $S_{c}=S,\forall c$ and $K_{s(c)}=K_{c},\forall s$. As an example, for n×n images using decimated wavelet transform, we would have $K_{s(c)}=n/2^{c}\times n/2^{c}$ and $S_{c}=3$ for all scales except for the last one where $S_{c}=4$. Moreover, the *k*th-coefficient in the *s*th-sub-band of the *c*th-scale for the *ℓ*th-coil will be denoted as $z_{csk\ell }$. Vector $z_{csk,:}\in C^{L}$ gathers the multi-channel coefficients ${\left(z_{csk\ell }\right)}_{1\leq \ell \leq L}$ at position *k*, sub-band *s* and scale *c*. Similarly, the larger vector $z_{cs,:}$ stacks the multi-position and multi-coil coefficients ${\left(z_{csk\ell }\right)}_{1\leq k\leq K_{c},1\leq \ell \leq L}$ at a given sub-band *s* of scale *c*. Last, vector ${}_{c,:}$ stacks the multi-band multi-position and multi-coil coefficients ${\left(z_{csk\ell }\right)}_{1\leq s\leq S,1\leq k\leq K_{c},1\leq \ell \leq L}$ at a given scale *c*.

The resolution of Problem (2) delivers *L* channel images ${\left({\hat{x}}_{\ell }\right)}_{1\leq \ell \leq L},\;\mathrm{stacked}\;\mathrm{in}\hat{X}$. Once Problem (2) is solved, all coil-specific MR images ${\left({\hat{x}}_{\ell }\right)}_{1\leq \ell \leq L}$ are combined using the square-root of the sum-of-squares (sSOS), ${\hat{x}}_{\mathrm{sSOS}}=\sqrt{\sum_{\ell =1}^{L}{∥{\hat{x}}_{\ell }∥}_{2}^{2}}$, to form a single *magnitude* image as usually done in parallel imaging [13]. The virtual coil method is also used to combine *phase information* across all coils [39] and get ${\hat{x}}_{s∠}$. Let us remark that Problem (2) is called *calibration-less*. It is in contrast with SENSE formulation which aims at directly reconstructing a single full FOV image $x\in C^{N}$ from multi-channel data ${\left(y_{\ell }\right)}_{1\leq \ell \leq L}$, assuming the sensitivity maps ${\left(S_{\ell }\right)}_{1\leq \ell \leq L}$ modulate the ground truth ***x*** as $x_{\ell }=S_{\ell }x$.

In what follows, we propose an efficient proximal optimization method to solve Problem (2).

### 2.3. Primal-Dual Optimization Algorithm

Problem (2) amounts to solving

(3) $$\hat{X}\in \underset{X\in C^{N\times L}}{\mathrm{argmin}}\left[f(X)+g(\Psi X)\right].$$

where we denote:

(4) $$\left(\forall X\in C^{N\times L}\right)\;f\left(X\right)=\sum_{\ell =1}^{L}{∥F_{M}x_{\ell }-y_{\ell }∥}_{2}^{2}/\left(2\sigma_{\ell }^{2}\right).$$

Function *f* belongs to $\Gamma_{0}\left(C^{N\times L}\right)$ and it is β-Lipschitz differentiable that is:

(5) $$\left(\forall X,X^{\prime }\in C^{N\times L}\right),\;∥\nabla f\left(X\right)-\nabla f\left(X^{\prime }\right){∥}_{2}\leq \beta {∥X-X^{\prime }∥}_{2},$$

with $\beta =\sum_{l=1}^{L}{\left(\sigma_{\ell }^{2}\right)}^{-1}\left||\right|F_{M}{\left||\right|}^{2}$. Moreover, function *g* belongs to $\Gamma_{0}\left(C^{N\times L}\right)$. We thus propose to make use of the proximal primal-dual algorithm ([40,41]) from Condat-Vú [42,43], which leads to Algorithm 1.
**Algorithm 1:** Condat-Vú algorithm
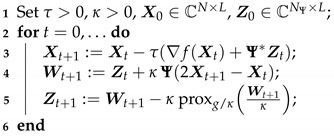


According to ([42], Theorem 3.1), the sequence ${\left(X_{t}\right)}_{t\in N}$ generated by Algorithm 1 weakly converges to a solution of Problem (3) as soon as $\frac{1}{\tau }-\kappa {\left\|\;|\Psi |\;\right\|}^{2}\geq \frac{\beta }{2}$. In practice, the hyper-parameters of this algorithm are set as follows: $\tau :=\frac{1}{\beta }$, $\kappa :=\frac{\beta }{2{\left\|\;|\Psi |\;\right\|}^{2}}$. Note that when **Ψ** is orthogonal, we get ‖|Ψ|‖=1. The main advantage of Algorithm 1 is that it does not involve the computation of ${\mathrm{prox}}_{g\circ \Psi }$. The latter does not usually have closed form, in particular when **Ψ** is overcomplete (e.g., undecimated wavelet transform), and would require the use of an inner iterative solver [44].

## 3. Octagonal Shrinkage and Clustering Algorithm for Regression

### 3.1. OSCAR Regularizer

#### 3.1.1. Definition

Let $z\in C^{p}$ with p≥1. OSCAR norm [30] is defined as follows:

(6) $$\Omega_{\lambda ,\gamma }\left(z\right)={\lambda ∥z∥}_{1}+\gamma \sum_{1\leq j<k\leq p}\max(|z_{j}\left|,\right|z_{k}\left|\right),$$

with λ and γ two positive hyper-parameters. The $\ell_{1}$-norm term in $\Omega_{\lambda ,\gamma }$ promotes the sparsity of ***z*** while the second term, corresponding to a pairwise *ℓ*_∞_-norm, encourages the equality of each entry pair in ***z***.

We now introduce the magnitude sorting operator $S_{p}:C^{p}\to C^{p}$ such that vector $S_{p}\left(z\right)={\left(S_{p}{\left(z\right)}_{j}\right)}_{1\leq j\leq p}$ contains the *p* entries of ***z*** sorted in decreasing order in magnitude, that is, such that

(7) $$|S_{p}{\left(z\right)}_{1}\left|\geq \right|S_{p}{\left(z\right)}_{2}\left|\geq \cdots \geq \right|S_{p}{\left(z\right)}_{p}|.$$

As pointed out in ([45], Section II. A.), OSCAR norm has a closed relation with the Ordered Weighted *ℓ*_1_ (OWL) norm defined below:

(8) $$\Theta_{w}(z)=\sum_{j=1}^{p}w_{j}\left|S_{p}{\left(z\right)}_{j}\right|,$$

with $w\in R_{+}^{p}$ a vector of hyper-parameters such that $w_{1}\geq \cdots \geq w_{p}\geq 0$. More precisely, OWL and OSCAR become equivalent if one sets the OWL weights as follows: $w_{j}=\lambda +\gamma \left(p-j\right)$ for j=1,…,p.

#### 3.1.2. Proximity Operator

Let $z\in C^{p}$. If ***z*** is equal to zero, then the proximity operator of the OWL norm at ***z*** is also equal to zero. Otherwise, it can be efficiently computed thanks to the following Algorithm 2 as shown in ([45], SectionIII A):
**Algorithm 2:** Proximity operator of the OWL norm._1_ Input: $z\in C^{p}/\left\{0\right\}$, $w\in R^{p}$;_2_ n=|z|/z;_3_ Let $P\in R^{p\times p}$ s.t. $S_{p}\left(n\right)=Pn$;_4_ Return ${\mathrm{prox}}_{\Theta_{w}}\left(z\right)=n⊙P^{⊤}\mathrm{PAV}\left(S_{p}\left(n\right)-w\right)$;

Hereabove, PAV refers to the Pool Adjacent Violator Algorithm [46] and ⊙ to the Hadamard product (i.e., element-wise multiplication). The proximity operator of OSCAR can thus be easily deduced by setting the appropriate value for mentioned above, so that OSCAR and OWL match together.

### 3.2. OSCAR-Based Image Reconstruction

Data acquired with a multi-coil array is highly correlated since the associated k-space samples are collected using the same readout, but with coil-specific and spatially overlapping sensitivity profiles. Sparsity-based inference in the highly correlated setup has been a well studied topic in data science [47,48]. In particular, it was pointed out that the $\ell_{1}$ regularization may fail when multiple block of variables are highly correlated as the solution tends to select one of those blocks. OSCAR regularization has been specifically designed to perform both shrinkage and variable selection [30,31,45]. It is thus well suited for multi-coil CS MR image reconstruction from highly correlated data.

In what follows, we propose four choices for function *g* in Problem (3) relying on OSCAR norm, with the aim to perform efficient calibration-less multi-coil MR image reconstruction. The main difference lies in the way the OSCAR norm is applied to the sparsifying decomposition $Z=\Psi X=[z_{1}\ldots z_{\ell }]\in C^{N_{\Psi }\times L}$ of the muti-coil image $X=[x_{1}\ldots x_{\ell }]\in C^{N\times L}$.

#### 3.2.1. Global OSCAR Regularization

The most straightforward way to implement OSCAR-based regularization consists in flattening all wavelet coefficients and thus discarding the multiscale and multi-coil structure (e.g., in scales, sub-bands, coefficients and coils) of ***Z***. For that reason, we call this version global OSCAR (g-OSCAR) regularization. The wavelet coefficients are stacked together, leading to a single but large vector with entries ${\left(z_{j}\right)}_{1\leq j\leq LN_{\Psi }}$, where we remind that $N_{\Psi }=S\sum_{c=1}^{C}K_{c}$ and $N_{\Psi }=N$ when **Ψ** is orthogonal. The g-OSCAR regularizer then reads:

(9) $$\begin{array}{cc} g_{g-OSCAR}\left(Z\right) & =\Omega_{\lambda ,\gamma }\left(Z\right)\\ \; & =\sum_{j=1}^{LN_{\Psi }}\lambda |z_{j}|+\gamma \sum_{1\leq j<k\leq LN_{\Psi }}\max\{|z_{j}\left|,\right|z_{k}\left|\right\}\\ \; & =\;\sum_{j=1}^{LN_{\Psi }}\left(\lambda +\gamma (LN_{\Psi }-j)\right)\left|S_{LN_{\Psi }}{\left(z\right)}_{j}\right|\;. \end{array}$$

#### 3.2.2. Scalewise OSCAR Regularization

We now propose a scalewise formulation, where OSCAR norm is applied to each specific scale *c* of the wavelet decomposition, hence to each vector $z_{c,:}$ separately. This leads to the so-called s-OSCAR regularizer:

(10) $$\begin{array}{cc} g_{s-OSCAR}\left(Z\right)\; & =\;\sum_{c=1}^{C}\Omega_{\lambda ,\gamma }\left(z_{c,:}\right)\\ \; & =\sum_{c=1}^{C}\sum_{j=1}^{LSK_{c}}\left(\lambda +\gamma (LSK_{c}-j)\right)\left|S_{LSK_{c}}{\left(z_{c,:}\right)}_{j}\right|\;, \end{array}$$

where vector $z_{c,:}$ gathers the $LSK_{c}$ wavelet coefficients across all coils in a specific scale c∈{1,…,C}. In that way, the wavelet coefficients can be clustered together regardless the sub-band they belong to, their position and their coil dependence. Thus, *C* sorting operations are required, each of them involving $LSK_{c}$ parameters. As the s-OSCAR regularization is separable by scales, the computation of its proximity operator can be performed efficiently using parallelization over scales.

#### 3.2.3. Subbandwise OSCAR Regularization

The present formulation applies OSCAR regularization to each specific subband of the wavelet decomposition, hence to each vector $z_{cs,:}$ separately. We then obtain the b-OSCAR regularizer:

(11) $$\begin{array}{cc} g_{b-OSCAR}\left(Z\right) & =\sum_{c=1}^{C}\sum_{s=1}^{S}\Omega_{\lambda ,\gamma }\left(z_{cs,:}\right)\\ \; & =\sum_{c=1}^{C}\sum_{s=1}^{S}\sum_{j=1}^{K_{c}L}\left(\lambda \;+\;\gamma (K_{c}L-j)\right)\left|S_{K_{c}L}{\left(z_{cs,:}\right)}_{j}\right|\;, \end{array}$$

where vector $z_{cs,:}$ gathers the $K_{c}L$ wavelet coefficients across all coils in a given subband *s* of scale *c*. Here again, the separability of the regularizer can be exploited for an efficient implementation of the proximity operator.

#### 3.2.4. Coefficientwise OSCAR Regularization

Finally, we propose applying the OSCAR norm to each wavelet coefficient separately and thus to each vector $z_{csk,:}$, leading to c-OSCAR regularization:

(12) $$\begin{array}{cc} g_{c-OSCAR}\left(Z\right) & =\sum_{c=1}^{C}\sum_{s=1}^{S}\sum_{k=1}^{K_{c}}\Omega_{\lambda ,\gamma }\left(z_{csk,:}\right)\\ \; & =\sum_{c=1}^{C}\sum_{s=1}^{S}\sum_{k=1}^{K_{c}}\sum_{\ell =1}^{L}\left(\lambda +\gamma (L-\ell )\right)\left|S_{L}{\left(z_{csk,:}\right)}_{\ell }\right|\;, \end{array}$$

where vector $z_{csk,:}$ gathers the *L* wavelet coefficients across all coils for coefficient *k* in sub-band *s* of scale *c*. This formulation is the closest to the usual application of the group-LASSO structured sparsity penalty [28] as it operates separately on each pixel in the transformed domain. However, instead of implicitly assuming a constant noise level across all coils by taking the $\ell_{2}$-norm, the c-OSCAR regularization allows a different weighting of the coils due to the sorting step. This makes the proposed regularization sensitive to space-varying noise levels.

## 4. Materials and Methods

We assessed the quality of the proposed calibration-less MR image reconstruction method, in terms of computational complexity and image quality through retrospective as well as prospective studies. We compared the four OSCAR-based formulations, described in Section 3, with state-of-the-art methods. These included calibration-less (CaLM [27], AC-LORAKS [49]) and self-calibrating ones ($\ell_{1}$-ESPIRiT [21]). AC-LORAKS was preferred to P-LORAKS [15] as it is less demanding from a computational viewpoint. All numerical experiments were conducted on 2D multi-coil k-space data even though the proposed framework could be extended to 3D imaging quite directly. Hence, we only report results on slices.

Importantly, retrospective analysis permits a direct evaluation and comparison of the behavior and performance of all methods on the same artificially under-sampled data set. This is the most secured way to compare reconstruction methods and allows extensive investigation by manipulating different sampling masks. However, prospective validation especially in the non-Cartesian setting is particularly interesting, as such readouts allow higher acceleration factors than Cartesian trajectories while preserving image quality. Moreover, it corresponds to actual accelerated data and then offers the possibility to look at how well the phase information is preserved on top of the quality of the magnitude contrast. CS reconstruction methods are barely compared in non-Cartesian imaging and even less in prospective acquisition scenarios. A significant advantage of this work is thus to permit such comparisons.

### 4.1. Reconstruction Parameters and Computational Complexity

In all the studies that follow, we ran Algorithm 1 until T=150 iterations, which appears sufficient for reaching convergence of the iterates. Moreover, we used for **Ψ** a Daubechies 4 orthogonal wavelet transform (OWT) with C=4 decomposition scales (i.e., $N_{\Psi }=N$). Note that MR image quality can be improved using redundant multiscale transforms (e.g., undecimated bi-orthogonal wavelet transforms or curvelets as shown in [50]) but this kind of decomposition significantly increases the memory load and computation time of the overall algorithm. Moreover, it does not change the actual comparisons of the four versions of OSCAR-norm regularization.

In Table 1, we summarize the numerical complexity associated with each OSCAR-norm regularization, the computing time required for evaluating both its proximity operator and running one full iteration of Algorithm 1. Whenever made possible, the parallel computation of the proximity step involved in the OSCAR-norm regularization was performed using joblib, a Python package that allows embarrassingly parallel computations (https://pypi.org/project/joblib/ (accessed on 10 February 2021)). The number of parallel threads that were used is indicated in Table 1. All experiments were run on a machine with 128 GB of RAM and an 8-core (2.40 GHz) Intel Xeon E5-2630 v3 Processor.

From the computational point of view, the g-OSCAR version is the less expensive as it requires a single evaluation of the proximity operator. Then, the b-OSCAR version is more amenable to parallelization (see Table 1) since the number of threads (16 in this case) matches the number of wavelet sub-bands for the decomposition. Further, we observe that c-OSCAR approach is most computationally demanding as this approach carries out OSCAR norm on every coefficient in wavelet domain. However, on preliminary studies, we did not see significant improvement in the image quality with c-OSCAR as compared to other methods. Hence, we did not carry out an extensive analysis of this method hereafter.

The overall algorithm was implemented in PySAP-MRI [51] (https://github.com/CEA-COSMIC/pysap-mri (accessed on 10 February 2021)), a python plugin to PySAP [52], an open source software written in Python and dedicated to sparse multiscale representation and analysis of images. For non-Cartesian reconstructions, the non-uniform fast Fourier transform was implemented on GPU through Python bindings for gpuNUFFT [53], which resulted in a large speedup.

### 4.2. Retrospective Study

For the purpose of retrospective study, we used raw multi-coil k-space from the brain fastMRI dataset [54,55]. In particular, we selected five slices of a given brain and we picked up two very different imaging contrasts, namely FLAIR and T1-weighted images. To understand the performance of our algorithm in wide range of experimental setups, we carried out retrospective studies in both Cartesian and non-Cartesian under-sampled acquisition scenarios. For Cartesian under-sampling, we limit the Cartesian mask to be a 1D variable density sampling along the phase encoding direction. The under-sampling factor was set to 4 (UF=4), which means that the data would be almost 4-fold accelerated (AF≃4) in time in prospective acquisitions. Indeed, a central area surrounding the k-space center was deterministically sampled to minimize coherent artifacts [19].

For non-Cartesian patterns, we carried out the retrospective under-sampling of the same MR images from the brain fastMRI dataset. We chose to rely on a 2D SPARKLING (Spreading Projection Algorithm for Rapid K-space samplING) [9,11], with an acceleration factor of AF=16 but a limited under-sampling factor of UF=1.66 as explained hereafter. To understand the difference between AF and UF, one has to recall the following relations: let N=n×n the number of pixel in the image domain with *n* the image dimension, assume that the data in k-space is composed of $n_{c}$ shots, each of them consisting of $n_{s}$ samples. Then, $\mathrm{AF}=\frac{n}{n_{c}}$ if we refer to Cartesian sampling as the full coverage corresponding to AF=1. In contrast, $\mathrm{UF}=\frac{n\times n}{n_{c}\times n_{s}}$. Hence, the acceleration factor reflects on how fast the scan is with respect to the Cartesian reference scan, while the under-sampling factor is a measure of how much the k-space is under-sampled with respect to fully sampled Cartesian k-space. In case of Cartesian studies, both AF and UF are approximately the same although the central portion of k-space is usually fully sampled to permit calibration of sensitivity maps in multi-coil acquisition. However, in non-Cartesian sampling scenarios, the trajectory can benefit from collecting a larger number of samples $n_{s}$ in a given shot with higher receiver sampling rates, thus allowing for reaching high AF with lower UF. In this study, we used n=320, $n_{c}=20$ and $n_{s}=3072$. As shown in [11], SPARKLING method generates physically plausible trajectories with improved robustness to gradient imperfections and is less prone to off-resonance artifacts compared to spiral trajectories. The resulting sampling schemes are known to reach higher image quality for a given scan time, compared to state-of-the art trajectories (e.g., spiral or radial).

The sampling patterns used for both the Cartesian and non-Cartesian cases are presented in Figure 3A,B respectively.

### 4.3. Prospective Study

We consider the reconstruction of an ex vivo human brain with an in plane resolution of 0.39×0.39 mm${}^{2}$ prospectively acquired on a 7T MR system (Magnetom Siemens Healthineers, Erlangen, Germany) using a L=32-channel coil (Nova Medical Inc., Washington, WA, USA). The acquisition parameters of the 2D Gradient Recalled Echo (GRE, that is, FLASH in Siemens-Healthineers systems) $T_{2}^{*}$ sequence were set as follows: FOV=200×200 mm${}^{2}$, TR=550 ms (for 11 slices), TE=30 ms, BW=100 kHz, $T_{\mathrm{obs}}=30.72$ ms (long readout) and FA = 25${}^{\circ }$ with in-plane resolution of 390 µm and slice thickness of 3 mm. Fully sampled Cartesian measurements were acquired and reconstructed into an image which served as ground truth using the same sequence parameters (matrix size: N=512×512 or n=512). As BW=100 kHz, each shot contains 3072 gradient steps. Further, we implemented oversampling along each shot by a factor of 2, so in the end we ended up with $n_{s}=6144$ samples. The SPARKLING trajectories were similar to (B) in Figure 3. These multi-shot trajectories were played by the head-only gradient system (AC84) installed on the 7 MR system. To analyze the performance of our algorithm at varying acceleration factors, we collected the k-space samples prospectively using 2D SPARKLING with varying AF from 8 ($n_{c}=64$) to 20 ($n_{c}=26$). These acceleration factors are matched to UF=0.66 (no under-sampling) and UF=1.66, respectively. In the present study we also rely on the structural similarity (SSIM) and peak signal-to-noise ratio (pSNR) scores for comparing image quality as the ex vivo human brain was fixed, hence preventing motion artifacts and reducing the variation in B0 inhomogeneity between scans.

### 4.4. Hyper-Parameters Search and Sensitivity

The hyper-parameters (λ,γ) were set using a grid search procedure so as to maximize the SSIM score [56] of the combined magnitude image ${\hat{x}}_{\mathrm{sSOS}}$. Herebelow we discuss this setting in the prospective case as it is potentially less robust.

**Figure 1:**
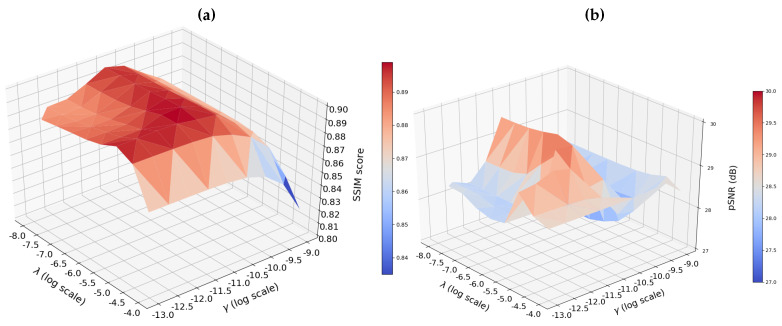
Map of (**a**) structural similarity (SSIM) (**b**) peak signal-to-noise ratio (pSNR) score as a function of hyperparameters (λ,γ) involved in OSCAR-band (b-OSCAR) regularization using 20-fold prospectively accelerated Sparkling sampling scheme.

We provide in Figure 1a an illustration of the range of variation of image quality as a function of the setting of (λ,γ). We show that the SSIM score reaches a plateau for a large range of parameters with a maximal and minimal values respectively reaching 0.899 and 0.822. In Figure 1b, we replicated the same analysis using the pSNR score for the same 20-fold SPARKLING prospectively accelerated data set. We found similar optimal values around $\left(\lambda ,\gamma \right)=\left(10^{-6.5},10^{-10.5}\right)$. In this case, the image quality remains quite constant as λ fluctuates and γ is fixed with a maximal and minimal values reaching respectively 29.52 dB and 27.33 dB. However, the pSNR value significantly drops off when γ departs from its optimal setting. Given this lack of robustness, all results presented in the following were obtained using the SSIM score as target metric for setting (λ,γ). This approach was retained both for OSCAR-norm regularization but also for its competitors (e.g., $\ell_{1}$-ESPIRIT, CaLM and AC-LORAKS).

### 4.5. Phase Processing

In the context of multi-coil acquisition, each coil’s image has its own specific magnetization phase that combines the organ magnetization and its own reception phase. The latter depends on the distance and position to the scanned organ. Therefore assessing the organ magnetization phase requires removing the relative coil phase. This is performed by creating a virtual reference coil that will be used to register the phase before combining the multiple coils as explained in [39].

**Figure 2:**
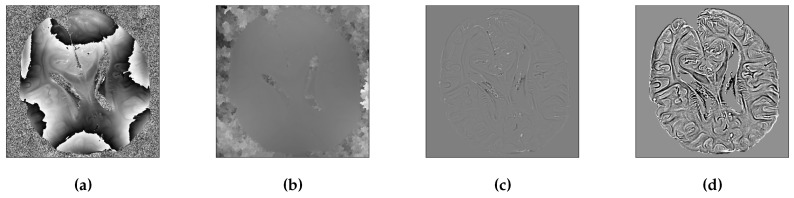
Phase processing for better visualization of the brain structure and hence easier comparisons. (**a**) Raw wrapped phase image. (**b**) Unwrapped phase image. (**c**) High pass filtered phase image, notice that the structures in the brain are more visible here. (**d**) Contrast stretching of 2nd and 98th percentiles of intensity values that permits contrast enhancement and improved visualization.

One significant aspect of OSCAR regularization that we will showcase in the next section is that it better preserves the phase information in the reconstructed image as compared to other methods. In T2*-weighted imaging, the phase of the reconstructed brain image is crucial for obtaining a susceptibility weighted image (SWI) (see [57]). To this end, it is important to rely on a clear and validated methodology that permits visual comparison of two phase images. For illustration purposes, we report in Figure 2a the key steps from the raw wrapped phase image to the post-processed unwrapped phase image. As shown in Figure 2a, the wrapped phase does not allow us to visualize the phase variations in various brain structures. Next, unwrapping the phase just flattens the whole image as illustrated in Figure 2b, thereby making differences between images harder to outline. For this reason, following the classical post-processing steps to obtain SWI images, we carried out a high-pass filtering of the phase image using a Hanning filter. As shown in Figure 2c, this step effectively cleans up the phase image and highlights the phase variations in different brain areas. Further, to enhance the structures in phase variation, we magnified the contrast intensity of the image between the 2nd and 98th percentile values of the histogram. For this, we used scikit-image (https://scikit-image.org/docs/dev/auto_examples/color_exposure/plot_equalize.html (accessed on 10 February 2021)). The result is depicted in Figure 2d.

Finally, for the sake of image phase comparison, we derived a quantification index. For this, we calculated the mean squared error (MSE) on hi-pass filtered phase image (Figure 2c) with respect to similar image of the Cartesian reference. This was done so that the metrics was robust to noise and wrapping artifacts. The MSE was carried out with a mask of the brain to have more reliable metrics.

## 5. Results

### 5.1. Retrospective Studies

**Figure 3:**
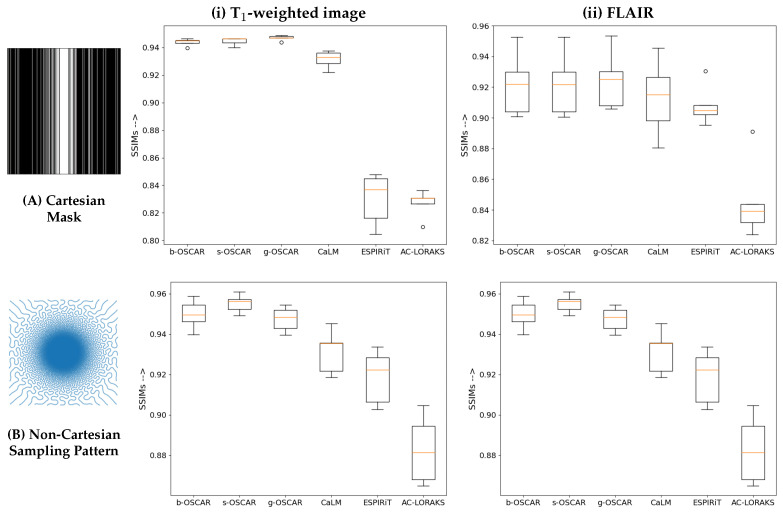
Retrospective results for Cartesian mask (**A**) and non-Cartesian undersampling pattern (**B**) on T1 weighted (**i**) and FLAIR (**ii**) images of the brain fastMRI data set. The results are presented in the form of box plots, computed over S=5 slices. From left to right in each boxplot, we compared subbandwise OSCAR (b-OSCAR), scalewise OSCAR (s-OSCAR), global OSCAR (g-OSCAR), CaLM, L1-ESPiRIT and AC-LORAKS, reconstruction methods.

**Figure 4:**
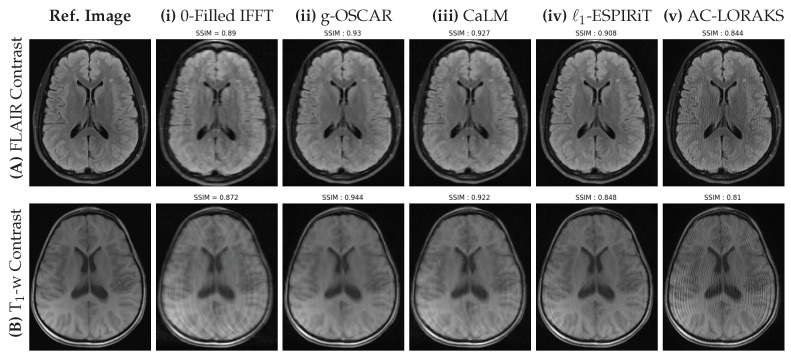
Retrospective results for a single slice of FLAIR (**top row**) and T1 weighted (**bottom row**) images of the brain fastMRI data set obtained using the Cartesian mask shown in Figure 3A with UF=4, corresponding to AF≃4. The fully sampled Cartesian reference and the different methods (Zero filled Inverse, g-OSCAR, CaLM, L1-ESPiRIT and AC-LORAKS) are shown from left to right and the SSIM scores are indicated to reflect the performance of each method.

**Figure 5:**
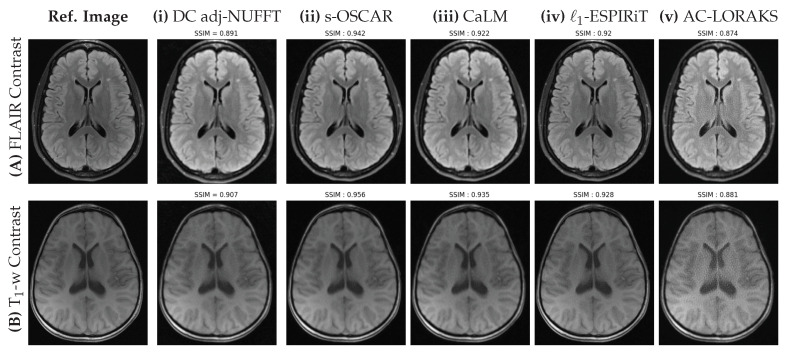
Retrospective results for a single slice of FLAIR (**top row**) and T1 weighted (**bottom row**) images of the brain fastMRI data set obtained using the Non-Cartesian sampling pattern shown in Figure 3B with AF=16 and UF=1.66. The fully sampled Cartesian reference and the different methods (Density Compensated (DC) adjoint NUFFT, s-OSCAR, CaLM, L1-ESPiRIT and AC-LORAKS) are shown from left to right and the SSIM scores are indicated to reflect the performance of each method.

We present all our retrospective results for all the slices (S=5) in the form of box plots of SSIM scores in Figure 3. We clearly observe that all the OSCAR based reconstructions outperform earlier state-of-the-art methods. Particularly, we observe that g-OSCAR performs the best in Cartesian retrospective study and s-OSCAR outperforms all methods for the non-Cartesian study. We proceed to present reconstructions on a single slice for all state-of-the-art methods and choose the best performing OSCAR method as a representative of our algorithm. However, it is worth noting that the differences associated with the different versions of OSCAR regularization are rather small. The Cartesian results are shown in Figure 4 and the non-Cartesian ones in Figure 5. Additionally, it is worth mentioning that we have added the zero-order solutions for both Cartesian and non-Cartesian under-sampling schemes (zero-filled IFFT and and DC Adjoint NUFFT, respectively).

Interestingly, in the retrospective Cartesian sampling scenario (cf. Figure 4), all methods perform better on the FLAIR contrast in comparison with the T1-weighted images which show severe aliasing artifacts. The zero-filled IFFT solution is severely blurred for both imaging contrasts. On top of the well visible artifacts on the L1-ESPiRIT and AC-LORAKS images, a loss in contrast is also observed for these methods on FLAIR images. In contrast, still on the FLAIR contrast CaLM and g-OSCAR report a limited amount of artifacts. The same under-sampling pattern appears more challenging on T1-weighting images as g-OSCAR is the only method which is capable to eradicate aliasing artifacts.

In Figure 5, our first observation is that all methods perform better in this non-Cartesian retrospective under-sampling study compared to the Cartesian one. The reason is quite obvious and due to the use of a lower under-sampling factor (UF=1.66) in this non-Cartesian setting as compared the 4-fold under-sampling in the Cartesian study. Although the UF is lower, this corresponds to a shorter scan time as the factor that mainly affects the acquisition time is the number of shots, irrespective of the number of samples per shot. This is a direct illustration of the benefit of implementing non-Cartesian encoding schemes. When comparing the different methods, it is worth noting the poor behavior of AC-LORAKS that reports lower SSIM scores and the wrong contrast. This results from the gridding step used before the application of the AC-LORAKS reconstruction method and the lack of density compensation as SPARKLING sampling implements variable density sampling. DC adjoint-NUFFT provides a better SSIM index than AC-LORAKS but faces similar contrast issues, especially on the FLAIR image. In contrast, all other competing techniques are pretty close and report SSIM scores larger than or equal to 0.92. Still the s-OSCAR provides the best contrast and less artifacts in the images.

### 5.2. Prospective Studies

**Figure 6:**
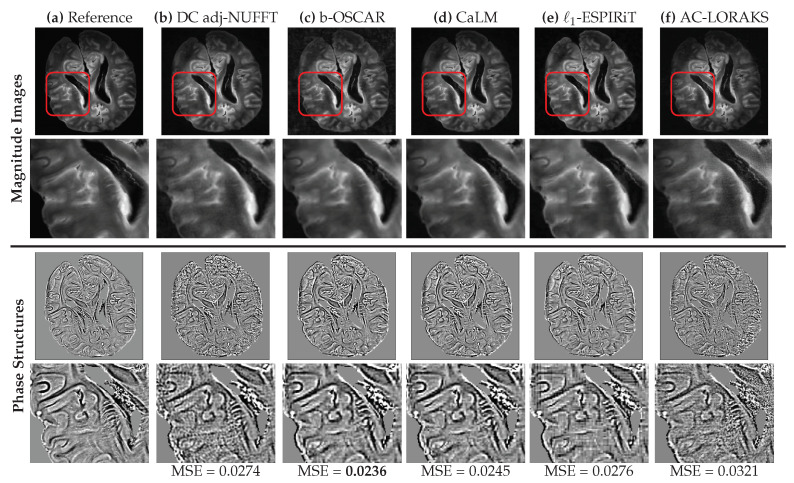
(**Top Row**) Reconstructed MR images (magnitude) from 20-fold accelerated SPARKLING acquisitions using different methods. The scan time for Cartesian reference was 4min41s while the scan time of accelerated SPARKLING was 15s. (**a**) Cartesian reference. (**b**) The density compensated adjoint of raw k-space data (DC adj-NUFFT). (**c**) Reconstruction based on the b-OSCAR formulation. (**d**) calibration-less reconstruction based on CaLM or group-LASSO regularization. (**e**) Self-calibrating $\ell_{1}$-ESPIRiT reconstruction. (**f**) Auto-calibrated (AC) LORAKS reconstruction. (**Second Row**) Respective zooms in the red frame. Reconstructed MR images. (**Third Row**) Enhanced structures in phase images obtained by the method described in Section 4.5 on the virtual coil reconstructions of each method. The respective MSE of the phase images with respect to Cartesian reference are also reported. (**Bottom Row**) Respective zooms to highlight details.

For prospective studies, we summarized the reconstruction results for varying acceleration factors in Table 2. We did not notice a huge difference between the different OSCAR versions in terms of SSIM score although we reported an improvement by 0.5 to more than 1dB on the PSNR values in favor of the sub-band-wise version (b-OSCAR). Hence, we will illustrate this version in the visual comparison in Figure 6. In terms of SSIM and pSNR scores, CaLM regularization remains close but below OSCAR, whether it is the sub-bandwise or scalewise version. This confirms that extending the group-LASSO penalization using a pairwise $\ell_{\infty }$-norm between channels instead of a global $\ell_{2}$-norm may be beneficial to account for spatially varying SNR across channels and to select the best coils in an adaptive manner across sub-bands or scales. The bottleneck in AC-LORAKS still lies in the gridding operation required to project the non-Cartesian samples onto the Cartesian grid. This step being not properly density compensated, we observed a degradation of image quality, hence confirming that AC-LORAKS may not be really compliant with non-Cartesian under-sampling.

Next, we compared the magnitude and phase information of the reconstructed slices. We selected the results corresponding to the most challenging acquisition scenario, namely the 20-fold prospectively accelerated SPARKLING data of the ex vivo human brain (scan time of 14 s for 11 slices instead of 4 min 42 s for the fully sampled Cartesian reference, see details in [11]). The corresponding images are presented in Figure 6. For the sake of completeness, we also show the density compensated adjoint NUFFT solution. First and foremost, on the magnitude images we observed that all methods perform pretty well even though AC-LORAKS shows more noise in the zoomed area marked by the red frame.

Second, we applied the post-processing to the phase image ${\hat{x}}_{s∠}$, that is, after the application of virtual coil reconstruction as explained in Section 4.5. We showed qualitatively and quantitatively that OSCAR better preserves phase information in comparison with DC Adjoint, CaLM, $\ell_{1}$-ESPIRiT and AC-LORAKS. The phase image yielded by AC-LORAKS is globally severely impaired likely due to the gridding operation while the one associated with $\ell_{1}$-ESPIRiT and DC adjoint NUFFT are really noisy in the zoomed area (red frame). Finally, as the visual comparison did not allow us to show a clear difference between OSCAR and CaLM regularization, the MSE index computed over the phase images helped us reveal that b-OSCAR has a slightly lower MSE score than the CaLM method.

## 6. Discussion

We formulated multi-coil CS image reconstruction as an inverse ill-posed problem and combined a classical $\ell_{2}$-norm data consistency term with OSCAR-norm penalty for regularization purposes. We instantiated four variants of OSCAR-norm regularization with different compromises between numerical complexity and modeling accuracy. Then, we relied on state-of-the-art convex nonsmooth optimization tools, namely the Condat-Vù algorithm with sound convergence properties to compute the global minimizer of the derived cost function. The main advantages of this algorithm is first that it can efficiently deal with analysis-based regularization which is known to provide better results than synthesis-based priors. Second, this algorithm is highly flexible, as it can be used the same way for a large range of penalizations and the parameters that control its convergence speed are easier to tune than those involved in ADMM (aka, split Bregman) methods [58].

The proposed method is also made of fully interpretable steps which make it reliable to perturbations in the data set (e.g., bad channel in the phased array coil with low SNR or small motion compensated in Non-Cartesian sampling due to variable density sampling) than could not be anticipated in recent deep learning approaches [59,60,61] proposed for medical image reconstruction. In the latter, sometimes a minor modification in the unseen test data set that was not met in the training and validation sets leads to major artifacts on reconstructed images [62].

The experimental validation was made on a wide range of acquisition setups. Retrospective studies were performed in both Cartesian and non-Cartesian setup using the brain fastMRI data set released in 2019 [54,55] and for two different imaging contrasts (T1-weighted and FLAIR). Complementary analyses were conducted on the T2 contrast and led to similar conclusions. A comparison with self-calibrating ($\ell_{1}$-ESPIRiT) and calibration-less (K-space based AC-LORAKS and wavelet based CaLM) methods was conducted. We further prospectively collected ex vivo human brain data at 7 Tesla using different acceleration factors. Image quality is similar for three regularization schemes, namely g-OSCAR, s-OSCAR and b-OSCAR penalties. However lowest algorithmic complexity and so computational cost was reported for b-OSCAR. Quantitative structural similarity scores show that OSCAR-based approaches slightly improved the overall image quality with SPARKLING trajectories compared to their competitors. On top of this improvement, phase information was better preserved for sparsity-based calibration-less reconstruction methods. This demonstrates the gain in robustness OSCAR-based reconstruction achieves compared to self-calibrating or gridded k-space based calibration-less techniques.

As for any regularized reconstruction approach, the performance of the proposed method depends on hyper-parameters. OSCAR penalization showed good stability over the setting of hyper-parameters, with a larger robustness range for λ compared to γ. In this work, both parameters have been set by maximizing the SSIM between a ground truth image reconstructed from a non-accelerated Cartesian acquisition and the image solution of Problem (2). Although this approach provided good results, it requires an additional lengthy Cartesian acquisition and leads to compare both different sampling schemes and reconstruction techniques. In the future, we will explore alternative automatic settings either based on (generalized) cross-validation [63] across slices for instance as a larger data set is necessary to implement this strategy, or on statistical inference (e.g., SURE estimator [63]), which requires the knowledge of noise statistics. The overall SSIM scores may also be improved using redundant wavelet transforms either considering undecimated decompositions or more sophisticated transforms such as Curvelet or Shearlet transforms [50,64]. Although the aforementioned approaches might improve MR image quality for each reconstruction, it could also increase the computation time.

The usual criticism of standard CS reconstruction methods is that they are highly computationally demanding compared to recent deep learning approaches. Although MRI vendors have shipped online CS reconstruction since 2017, their product is most often limited to low acceleration factors and to Poison Disk sampling. Recently we have extended online CS reconstruction to various sampling patterns and single-channel receiver coil for 2D imaging [65]. The main advantage of this algorithm is that it allows the physician to visualize the final reconstructed image by the end of acquisition without any supplementary delay. The proposed calibration-less reconstruction framework may be ideal to extend this online technique to the multi-coil setup as it can run online reconstruction over multiple coils without any prior knowledge on the sensitivity maps or any deviation of these maps along the scan. Also, both algorithms rely on the same primal-dual algorithm, hence this extension is quite straightforward.

The proposed method can be easily extended to 3D imaging and used as such in isotropic high-resolution susceptibility weighted imaging (SWI). In this context, the scan time is longer and SWI acquisitions in clinical routine will benefit from non-Cartesian highly accelerated encoding schemes to reach 600 m in 2 min 30 s [12]. Noticeably, in SWI imaging, post-processing is applied to phase information in order to reveal potential alterations of the microvascular brain network. The fact that our calibration-less regularized reconstruction better preserves phase information is thus an asset for its utilization in SWI.

## 7. Conclusions

In this paper, we have proposed a novel calibration-less MR image reconstruction method that relies on OSCAR-norm regularization. We have implemented four variants and shown that the global, scalewise and subbandwise provide very close results in terms of image quality with a best compromise in numerical complexity for the subbandwise version. All these variants fit within the same primal-dual optimization algorithm that converges to the global optimizer given the convexity of the cost function and the technical conditions met on the primal and dual step sizes. Retrospective studies conducted on the multi-contrast brain fastMRI data set have shown the major image quality improvement. This is a direct benefit of the proposed adaptive structure sparsity regularization compared with both self-calibrated ($\ell_{1}$-ESPIRiT) and calibration-less (CaLM, AC-LORAKS) reconstruction methods. To adequately cover a wide range of acquisition scenarios, these results have been obtained both on highly undersampled (Cartesian encoding) and accelerated (non-Cartesian encoding) k-space data collected over a multi-channel coil. Complementary prospective studies conducted on 7T ex vivo human brain data have led to similar conclusions with a significant gain on the recovery of phase information for OSCAR regularization. Overall, this work has shown that sensitivity maps no longer need to be estimated in multi-coil acquisition, a critical step to achieve online and robust CS reconstruction to multiple sources of artifacts (e.g., motion), especially when addressing massively accelerated high-resolution functional MRI.

## Figures and Tables

**Table 1:** Numerical complexity and parallelization capacity of OSCAR-norm regularizations using Daub. 4 OWT and (N,C,L)=(640×320,4,16) for MRI reconstruction. The computation times of other state of art methods (CaLM, $\ell_{1}$-ESPIRiT and AC-LORAKS) are also mentioned below.

	Proximity Numerical Complexity	Computation Time Per Prox. (S)	Parallelization	Computation Time Per Iter. (S)
g-OSCAR	$O(LN_{\Psi }\log\left(LN_{\Psi }\right))$	0.334	N.A.	2.894
s-OSCAR	$O(\sum_{c=1}^{C}LK_{c}S_{c}\log\left(LS_{c}K_{c}\right))$	1.005	*C*	6.711
b-OSCAR	$O(\sum_{c=1}^{C}LK_{c}S_{c}\log\left(LK_{c}\right))$	3.094	CS	4.418
c-OSCAR	$O(N_{\Psi }L\log L)$	159.75	$N_{\Psi }$	161.13
CaLM				1.944
$\ell_{1}$-ESPIRiT				4.360
AC-LORAKS				2.516

**Table 2:** Comparison of different OSCAR-norm regularizations with $\ell_{1}$-ESPIRiT, CaLM and AC-LORAKS. The hyper-parameters were set to maximize the SSIM score. Best image quality metrics computed per row appear in bold font. At most, three scores are outlined on each row.

AF	g-OSCAR		s-OSCAR		b-OSCAR		CaLM		$\ell_{1}$-ESPIRiT		AC-LORAKS	
	SSIM	pSNR	SSIM	pSNR	SSIM	pSNR	SSIM	pSNR	SSIM	pSNR	SSIM	pSNR
8	0.923	30.52	0.925	31.66	**0.926**	**31.68**	0.921	30.51	0.911	27.82	0.894	26.09
10	0.920	29.21	0.921	29.62	**0.922**	**30.28**	0.921	29.54	0.906	26.58	0.897	26.23
12	0.916	28.81	**0.918**	28.40	**0.918**	**29.78**	0.917	29.05	0.904	27.17	0.893	26.25
15	0.912	29.28	0.912	29.05	**0.913**	**29.52**	0.912	28.87	0.900	26.29	0.884	25.94
20	**0.899**	29.12	0.896	28.35	**0.899**	**29.52**	0.897	28.59	0.885	26.48	0.753	25.52

## Data Availability

The fastMRI data is available publicly at https://fastmri.org/ (accessed on 10 February 2021).
